# TRPM2 Deficiency Attenuates Allergic Rhinitis‐Like Inflammation With Altered Ca^2+^‐NFAT Signaling, Treg Responses, and sIgE Production

**DOI:** 10.1002/iid3.70490

**Published:** 2026-07-12

**Authors:** Zhenke Huang, Jianping Fan, Xinxin Shan, Juntao Su, Lixin Zhu, Lijing Peng

**Affiliations:** ^1^ Department of Otolaryngology Shanghai University of Medicine and Health Sciences Affiliated Zhoupu Hospital Shanghai China; ^2^ Department of Orthopedics Shanghai University of Medicine and Health Sciences Affiliated Zhoupu Hospital Shanghai China

**Keywords:** allergic rhinitis, Ca^2+^‐NFAT pathway, cytokines, IgE, Tregs

## Abstract

**Background:**

Allergic rhinitis (AR) is a prevalent chronic inflammatory condition characterized by nasal itching, sneezing, and congestion, significantly impairing patients' quality of life. Despite the availability of various therapeutic options, treatment efficacy remains suboptimal for certain patients, and long‐term use may be accompanied by adverse effects.

**Objective:**

This study examined the role of transient receptor potential melastatin 2 (TRPM2) in AR‐like inflammation, focusing on its associations with T cell functionality, Th2 inflammatory responses, Treg/Th17 balance, and upstream Ca^2+^‐NFAT signaling pathways.

**Methods:**

Using TRPM2 knockout and WT mice within an ovalbumin‐induced AR model, this research integrated behavioral assessments, histopathological analyses, immunological assays, qPCR, and Western blotting to evaluate the implications of TRPM2 deficiency for clinical symptoms, inflammatory responses, immune cell differentiation, and related signaling pathways.

**Results:**

TRPM2 knockout mice exhibited reduced clinical symptoms and nasal inflammation, lower serum OVA‐specific IgE levels, and reduced expression of key inflammatory cytokines, including IL‐4, IL‐5, and IL‐33. Furthermore, TRPM2 deficiency was associated with expansion of Treg cells, reduced Ca^2+^ influx, decreased NFATc1 nuclear translocation, and lower IL‐2 production. Although IL‐17 expression was reduced, the decrease in Th17 cell frequency did not reach statistical significance.

**Conclusion:**

These findings suggest that TRPM2 participates in OVA‐induced AR‐like inflammation through immune and Ca^2+^‐NFAT‐associated mechanisms, while the mechanistic and translational implications require cautious interpretation.

## 1 Introduction

Allergic rhinitis (AR) represents a prevalent and chronic inflammatory disorder of the upper respiratory tract, characterized by symptoms such as nasal itching, sneezing, rhinorrhea, and nasal congestion. These manifestations considerably impair patients' quality of life and contribute to substantial socioeconomic burden worldwide [1]. In recent decades, the incidence and prevalence of AR have shown a marked increase, largely attributed to environmental changes, urbanization, and heightened allergen exposure, rendering AR a critical global public health issue [2, 3]. Epidemiological surveys indicate that AR affects up to 10%–40% of the global population, with rising trends observed across diverse age groups and geographic regions [4]. Notably, the chronic and recurrent nature of AR not only results in direct morbidity but is also associated with comorbidities such as asthma, sinusitis, and sleep disturbances, further amplifying its clinical relevance [5].

The underlying pathogenesis of AR is multifaceted, involving a complex interplay of genetic predisposition, environmental triggers, and immune dysregulation. Central to AR is the aberrant activation of type 2 helper T (Th2) cells, which orchestrate eosinophilic inflammation and promote immunoglobulin E (IgE) production following allergen exposure [6, 7]. This Th2‐skewed immune response is accompanied by an imbalance involving regulatory T (Treg) cells, Th17 cells, and their respective cytokine profiles, contributing to the persistence and severity of AR [4]. At the molecular level, several signaling pathways, including nuclear factor kappa B (NF‐κB), mitogen‐activated protein kinases (MAPKs), and STAT3, have been implicated in the amplification of inflammatory cascades and the perpetuation of allergic inflammation [8, 9]. Although established pharmacotherapies such as antihistamines, intranasal corticosteroids, and leukotriene receptor antagonists can provide symptomatic relief, their long‐term effectiveness is limited in a subset of patients, and adverse effects may undermine patient compliance [10, 11]. Thus, upstream regulatory mechanisms underlying AR pathogenesis remain important candidate areas for investigation.

AR is increasingly recognized as a syndrome driven by diverse underlying biological mechanisms, or endotypes, rather than a single disease entity. These endotypes can be broadly categorized based on distinct inflammatory pathways, such as predominant type 2 inflammation involving Th2 cells, eosinophils, and immunoglobulin E (IgE), or other non‐type 2 responses. The experimental model employed in this study, induced by ovalbumin (OVA) sensitization, recapitulates key features of a type 2‐high AR endotype characterized by Th2 activation, eosinophilic inflammation, and elevated allergen‐specific IgE production. While this endotype represents an important component of clinical AR, it does not fully capture the heterogeneity of human disease. The pathogenesis of this specific endotype involves particular emphasis on Th1/Th2 balance, IgE synthesis, and the regulation of local inflammatory responses [7]. Nevertheless, current research is predominantly centered on downstream immune effectors, while less attention has been afforded to the upstream signaling pathways that govern immune cell activation and functional polarization. Of special interest are calcium (Ca^2+^) signaling pathways, which regulate an array of immune cell processes, including activation, cytokine secretion, and differentiation [12]. Among the Ca^2+^‐permeable ion channels, the transient receptor potential melastatin 2 (TRPM2) channel has emerged as an important mediator of oxidative stress responses and immune modulation across several disease contexts [13, 14]. TRPM2 is activated by adenosine diphosphate ribose (ADPR) and reactive oxygen species (ROS), resulting in Ca^2+^ influx that can shape cellular responses to inflammation [12]. While the involvement of TRPM2 in diverse inflammatory and metabolic disorders is increasingly recognized, its function within the immunopathology of AR remains insufficiently characterized.

In light of these considerations, the present study was designed to examine the role of TRPM2 in OVA‐induced AR‐like inflammation. Unlike prior investigations that have largely focused on Th2‐mediated responses or downstream signaling molecules, this research evaluates the association of TRPM2 deficiency with T cell function, Th2 inflammation, and immune tolerance. Furthermore, the study explores the relationship between TRPM2 and the Treg/Th17 axis, as well as Ca^2+^‐dependent nuclear factor of activated T cells (NFAT) signaling, providing a more integrated view of the immunoregulatory landscape in this murine AR model [12].

## 2 Materials and Methods

### 2.1 Animals and Allergic Rhinitis Model Establishment

Both wild‐type (WT) and TRPM2^−/−^ mice were used in this study. An ovalbumin (OVA)‐induced murine model of allergic rhinitis (AR) was established as previously described with minor modifications. Briefly, at systemic sensitization phase, beginning on day 0, mice were intraperitoneally injected with a suspension containing 50 µg of OVA and 5 mg of aluminum hydroxide Al(OH)3 as an adjuvant in 1 mL of sterile saline. Sensitization was performed every other day for a total of seven injections. For local challenge phase: from day 14, mice were stimulated by daily intranasal challenge with 5% OVA (20 μL) for 7 consecutive days from day 14. The animal experiments in this study were approved by the Medical Ethics Committee of Shanghai University of Medicine and Health Sciences Affiliated Zhoupu Hospital (Approval No. 2024‐C‐212).

### 2.2 Behavioral Assessment

Behavioral symptoms (sneezing, nasal rubbing, and nasal discharge) were recorded for 30 min immediately after the last challenge by two observers blinded to genotype. A standardized scoring system (Table 1) was used, and a total score > 5 was considered successful AR model establishment.

**TABLE 1:** Behavioral assessment criteria for mice.

Scoring/score	Sneezing	Nasal scratching/frequency	Nasal discharge
1	< =3	< =4	Mild nasal discharge, confined to the anterior nares
2	4–10	Vigorous scratching at the nose	Moderate nasal discharge exceeding the anterior nares
3	> =11	Rubbing of the head and body against surfaces	Profuse nasal discharge covering the fur around the mouth and nose

### 2.3 Histopathological Analysis

Twenty‐four hours after the final OVA challenge, mouse heads were collected and fixed in 4% paraformaldehyde for 48 h. Following fixation, tissues were decalcified in 10% EDTA (pH 7.4) for 2–3 weeks, embedded in paraffin, and sectioned sagittally at 4 μm thickness. Sections were stained with hematoxylin and eosin (H&E) according to standard protocols for evaluation of epithelial integrity, inflammatory cell infiltration, and glandular hyperplasia. Histopathological assessment was performed by two independent observers blinded to experimental groups using a light microscope.

### 2.4 Measurement of Serum OVA‐sIgE by ELISA

Venous blood was collected via eyeball enucleation and allowed to clot at room temperature for 30 min. Serum was separated by centrifugation at 3000 rpm for 10 min at 25°C. The ELISA kit components and samples were equilibrated to room temperature for 30 min. OVA‐sIgE standard was reconstituted to 500 pg/mL and then subjected to two‐fold serial dilution. According to the manufacturer's protocol, 100 μL of standards (from low to high concentration) or test samples (in duplicate) were added to appropriate wells. The plate was covered and incubated at 37°C for 2 h, followed by three washes with 300 μL wash buffer. Then, 100 μL streptavidin‐HRP working solution was added to each well, and the plate was incubated at room temperature for 1 h. After three additional washes, 100 μL chromogenic substrate was added and developed in the dark for 30 min. The reaction was terminated with 100 μL stop solution, and the yellow color development indicated completion.

### 2.5 RNA Extraction and cDNA Synthesis

Total RNA was extracted from approximately 30 mg of nasal mucosal tissue using TRIzol reagent (Invitrogen). Briefly, the tissue was homogenized in liquid nitrogen and mixed with 1 mL of TRIzol. After incubation at room temperature for 5 min, 200 μL of chloroform was added, and the mixture was centrifuged at 12,000 × g for 15 min at 4°C. The aqueous phase was collected and mixed with an equal volume of isopropanol. The RNA precipitate was washed twice with 75% ethanol, air‐dried, and dissolved in RNase‐free water. RNA concentration was measured using a spectrophotometer and adjusted to 500 ng/μL. cDNA was synthesized from 500 ng of total RNA using the PrimeScript RT Master Mix (Takara) in a 10 μL reaction volume according to the manufacturer's protocol.

### 2.6 Quantitative PCR Analysis

The mRNA expression levels of IL‐4, IL‐5, and IL‐33 were determined by quantitative real‐time PCR using TB Green Premix Ex Taq (Takara) on a QuantStudio 5 Real‐Time PCR System (Applied Biosystems). The primer sequences used are listed in Table 2. The PCR protocol consisted of initial denaturation at 95°C for 30 s, followed by 40 cycles of 95°C for 5 s and 60°C for 34 s. Melting curve analysis was performed to verify amplification specificity. All samples were run in duplicate, and gene expression levels were normalized to β‐actin using the 2^–ΔΔCt method.

**TABLE 2:** The primers of target genes.

Primers	Primer sequences
IL‐4 (forward)	5′‐CCCCAGCTAGTTGTCATCCTG‐3′
IL‐4 (reverse)	5′‐CAAGTGATTTTTGTCGCATCCG‐3′
IL‐5 (forward)	5′‐CTCTGTTGACAAGCAATGAGACG‐3′
IL‐5 (reverse)	5′‐TCTTCAGTATGTCTAGCCCCCTG‐3′
IL‐33 (forward)	5′‐AAGTACAGCATTCAAGACCAGC‐3′
IL‐33 (reverse)	5′‐GGTCTTCTGTTGGGATCTTCTTATT‐3′
Foxp3 (forward)	5′‐GAAAGCGGATACCAAATGA‐3′
Foxp3 (reverse)	5′‐CTGTGAGGACTACCGAGCC‐3′
β‐actin (forward)	5′‐CCCATCTACGAGGGCTAT‐3′
β‐actin (reverse)	5′‐TGTCACGCACGATTTCC‐3′

### 2.7 Isolation of Splenic CD4^+^ T Cells

Spleens were harvested from WT and TRPM2^−/−^ mice (*n* = 5 per group) following OVA sensitization and challenge. Single‐cell suspensions were prepared by mechanical dissociation through a 70‐μm cell strainer. Red blood cells were lysed using ACK lysis buffer. Naïve CD4^+^ T cells were then isolated by negative selection using a commercial mouse CD4^+^ T Cell Isolation Kit (Cat. No. 130‐104‐454, Miltenyi Biotec, Bergisch Gladbach, Germany), according to the manufacturer's instructions. Cell viability was confirmed to be > 95% by Trypan Blue exclusion.

### 2.8 Ca^2+^ Flux Assay by Flow Cytometry

Intracellular Ca^2+^ flux was measured using the fluorescent Ca^2+^ indicator Fluo‐4 AM (Thermo Fisher Scientific, USA, catalog number F14201). The isolated CD4^+^ T cells were resuspended in pre‐warmed (37°C) assay buffer (Hanks' Balanced Salt Solution, HBSS, supplemented with 20 mM HEPES and 0.1% BSA) at a density of 5 × 10^6^ cells/mL. Cells were loaded with 4 μM Fluo‐4 AM and incubated for 30 min at 37°C in the dark. Following incubation, cells were washed twice with assay buffer to remove excess dye and resuspended in fresh buffer. The cells were then rested for 15 min at room temperature before analysis. Briefly, baseline fluorescence was recorded for approximately 60 s to establish a stable signal. Subsequently, cells were stimulated by adding a pre‐optimized final concentration of 5 μg/mL each of anti‐CD3 (clone 145‐2C11, Cat. No. 100301) and anti‐CD28 (clone 37.51, Cat. No. 102119) antibodies, which were obtained from BioLegend (San Diego, CA, USA). Fluorescence data (excitation: 488 nm, emission: 530/30 nm) were collected continuously for at least 5 min post‐stimulation. Data were analyzed using *FlowJo v10.8* software, and the mean fluorescence intensity (MFI) over time was plotted.

### 2.9 Western Blot Analysis

Protein expression was analyzed by Western blotting. Tissue or cell samples were lysed in RIPA buffer supplemented with protease and phosphatase inhibitors. The protein concentration was determined using a BCA protein assay kit. Equal amounts of protein (20–30 μg) were separated by 10% SDS‐PAGE and transferred onto PVDF membranes. The membranes were blocked with 5% non‐fat milk in TBST for 1 h at room temperature and then incubated with primary antibodies at 4°C overnight. After washing with TBST, the membranes were incubated with HRP‐conjugated secondary antibodies for 1 h at room temperature. Protein bands were visualized using enhanced chemiluminescence (ECL) substrate and detected with a chemiluminescence imaging system. Lamin B1 was used as the internal control of nucleus for normalization. The primary antibodies used were shown as below: NFATc1 (Cat. No. 5861, Cell Signaling Technology); IL‐2 (Cat. No. 12239, Cell Signaling Technology); and Lamin B1 (Cat. No. ab133741, Abcam).

## 3 Results

### 3.1 TRPM2 Deficiency Alleviates Allergic Rhinitis Symptoms and Nasal Tissue Inflammation

**FIGURE 1:**
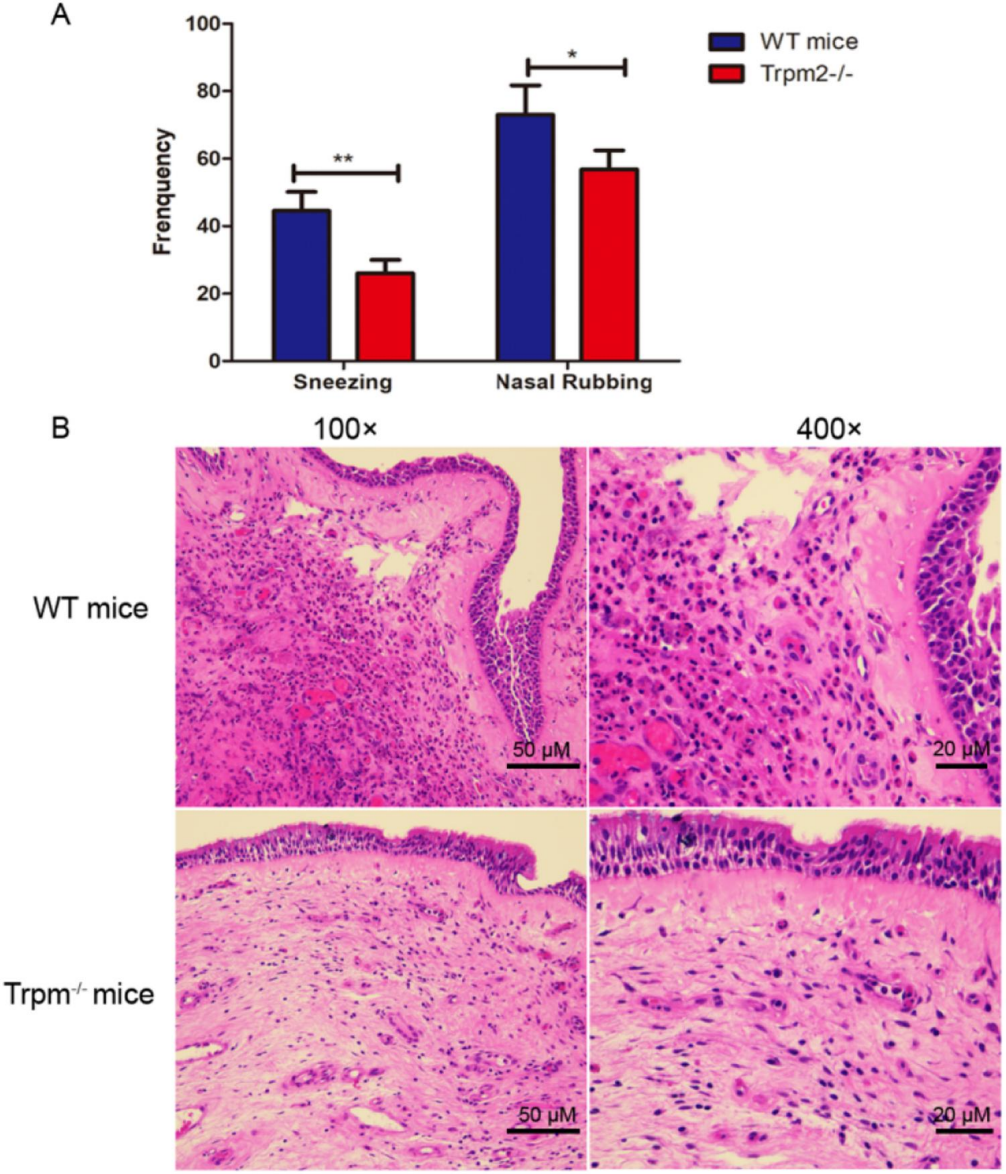
TRPM2 deficiency alleviates allergic rhinitis symptoms and nasal inflammation. (A) Behavioral assessment of sneezing and nasal rubbing frequencies in WT and TRPM2^−/−^ mice (*n* = 8) over 10 min after the final OVA challenge. Data are presented as mean ± SEM. *p < 0.05, **p < 0.01 vs. WT group (Student's *t*‐test). (B) Representative photomicrographs of H&E‐stained nasal mucosal sections. The WT group showed disrupted epithelium, substantial inflammatory cell infiltration, and glandular hyperplasia. The TRPM2^−/−^ group exhibited preserved epithelial integrity and reduced inflammation. (original magnification, ×100, ×400).

To investigate the role of TRPM2 in allergic rhinitis (AR) pathogenesis, we first compared the clinical manifestations and histopathological changes between WT and TRPM2^−−^ mice following OVA sensitization and challenge. Behavioral assessments revealed that TRPM2^−/−^ mice exhibited significantly reduced AR symptoms compared to WT controls (Figure 1A). The frequency of sneezing episodes decreased by 48.7% (WT: 44.5 ± 5.6 vs. TRPM2^−/−^: 26.0 ± 3.9 per 10 min, *p* < 0.01), while nasal rubbing events were reduced by 28.3% (WT: 72.9 ± 8.8 vs. TRPM2^−/−^: 56.8 ± 5.6 per 10 min, *p* < 0.05). Histopathological analysis of nasal mucosa by H&E staining demonstrated observable differences between groups (Figure 1B). WT mice displayed characteristic AR pathology, including disrupted epithelial integrity with ciliary loss, marked submucosal infiltration by eosinophils and mononuclear cells, and prominent glandular hyperplasia in the lamina propria. In contrast, TRPM2^−/−^ mice showed well‐preserved epithelial structure, reduced inflammatory cell infiltration, and minimal glandular changes. These results indicate that genetic ablation of TRPM2 attenuated AR‐associated clinical symptoms and tissue inflammation in this OVA‐induced model.

### 3.2 TRPM2 Deficiency Attenuates OVA‐Specific IgE Production

**FIGURE 2:**
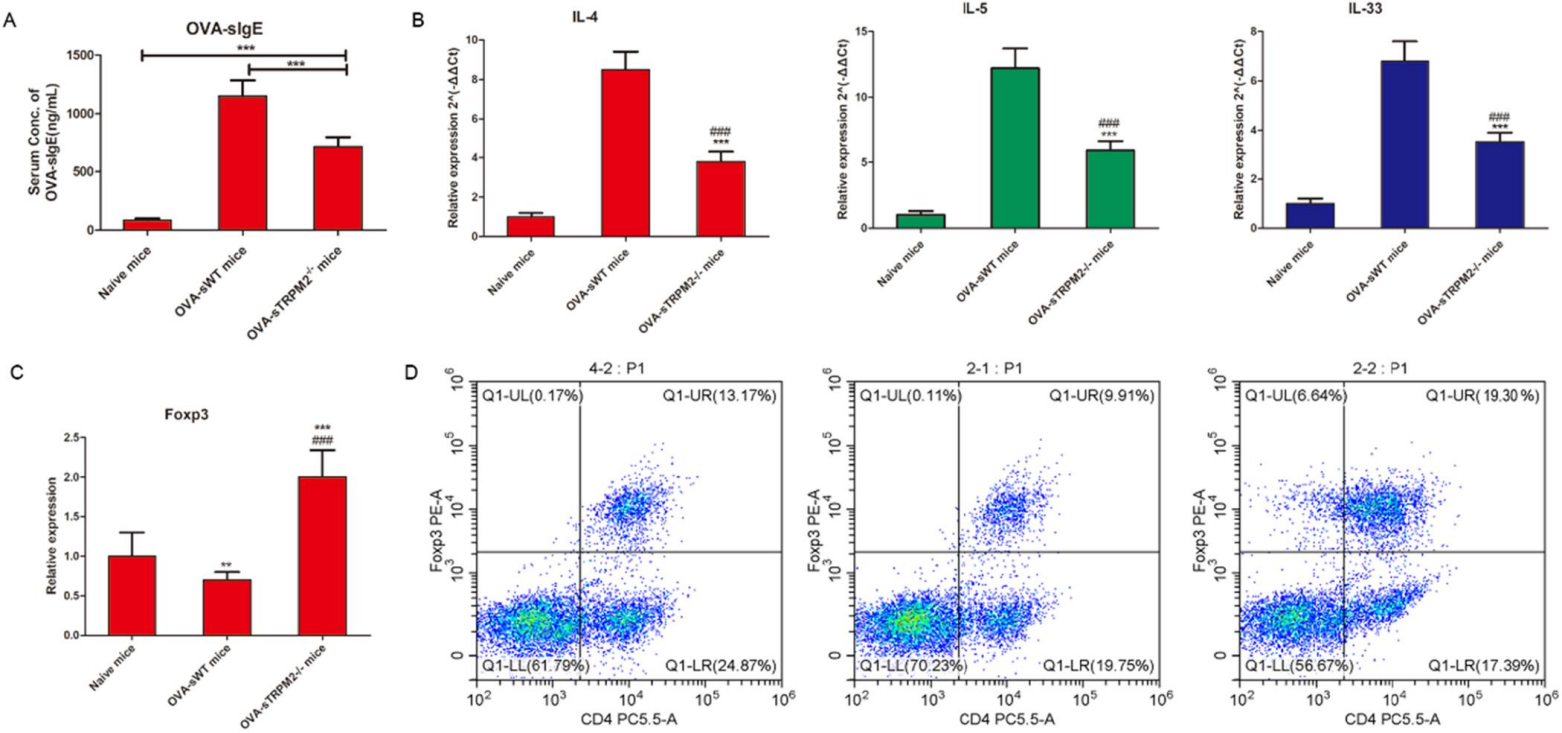
TRPM2 deficiency alleviates allergic responses by modulating humoral immunity, Th2 inflammation, and Treg responses. (A) Serum levels of OVA‐specific IgE in naïve, OVA‐sensitized wild‐type (WT), and OVA‐sensitized TRPM2^−/−^ mice, as measured by ELISA. TRPM2 deficiency significantly attenuated the OVA‐specific IgE response. ***p < 0.001 vs. OVA‐sensitized WT group; ^###^ *p* < 0.001 vs. naïve group. (B) Relative mRNA expression of Th2 cytokines (IL‐4 and IL‐5) and the alarmin IL‐33 in the nasal mucosa, determined by qPCR. The OVA‐induced upregulation of these mediators was significantly reduced in TRPM2^−/−^ mice. *p < 0.05, ***p < 0.001 vs. OVA‐sensitized WT group; ^#^ *p* < 0.05, ^##^ *p* < 0.01 vs. naïve group. (C) Relative mRNA expression of the Treg master transcription factor Foxp3 in splenic tissue. TRPM2 deletion increased Foxp3 expression. ***p < 0.001 vs. OVA‐sensitized WT group; *p < 0.05, ^###^ *p* < 0.001 vs. naïve group. (D) Representative flow cytometry plots of the frequency of CD4^+^Foxp3^+^ regulatory T (Treg) cells in the spleen.

To further elucidate the immunomodulatory effects associated with TRPM2 in allergic rhinitis pathogenesis, we performed immunological characterization in OVA‐sensitized mice. As shown in Figure 2A, ELISA measurement revealed low levels of OVA‐sIgE in naïve mice (85.5 ± 15.2 ng/mL), which were markedly elevated in OVA‐sensitized WT mice (1150.0 ± 135.5 ng/mL, *p* < 0.001), confirming successful model establishment (Figure 2A). TRPM2 deficiency significantly attenuated this OVA‐specific IgE response, reducing serum levels to 712.5 ± 88.3 ng/mL (*p* < 0.01 vs. OVA‐sensitized WT group). The IgE level in TRPM2^−/−^ mice remained significantly higher than in naïve controls (*p* < 0.001), indicating that TRPM2 deficiency partially, rather than completely, attenuated the humoral immune response.

### 3.3 TRPM2 Deficiency Suppresses IL‐4, IL‐5, and IL‐33 Production in the Nasal Mucosa

We further examined the expression of key Th2 cytokines in the nasal mucosa. qPCR analysis showed that the mRNA levels of IL‐4 and IL‐5 were increased in OVA‐sensitized WT mice compared with naïve controls (*p* < 0.001), as was the epithelial‐derived alarmin IL‐33 (Figure 2B). In TRPM2^−/−^ mice, this Th2‐skewed inflammatory response was significantly mitigated, with IL‐4 and IL‐5 expression reduced (*p* < 0.01 vs. WT OVA group) (Figure 2B), accompanied by a decrease in epithelial‐derived IL‐33 (*p* < 0.05). These results suggest that TRPM2 deficiency is associated with lower Th2 cytokine expression and attenuated epithelial‐immune inflammatory signaling.

### 3.4 TRPM2 Deletion Restores Immunosuppressive Function by Increasing CD4+Foxp3+ Tregs

The immunoregulatory capacity was reduced in AR mice but enhanced by TRPM2 deletion. Compared to naïve controls, splenic Foxp3 mRNA expression was significantly suppressed in WT OVA mice (*p* < 0.05). In contrast, TRPM2^−/−^ mice exhibited increased Foxp3 expression relative to naïve levels (*p* < 0.001) and WT OVA controls (*p* < 0.001, Figure 2C). A parallel trend was observed in Treg populations. The frequency of splenic CD4+Foxp3+ Tregs was significantly lower in the WT OVA group than in naïve mice (*p* < 0.05). TRPM2 deficiency rescued this suppression and increased the Treg compartment, significantly exceeding both the WT OVA and naïve groups (*p* < 0.001 and *p* < 0.01, respectively; Figure 2D). These findings support an association between TRPM2 deficiency and amelioration of AR‐associated immune dysregulation through reduced Th2 responses, attenuation of epithelial‐derived inflammatory signals, and enhancement of Treg‐mediated immunoregulation.

### 3.5 TRPM2 Deficiency Is Associated With Attenuated Ca^2+^‐NFAT Signaling During T Cell Activation

**FIGURE 3:**
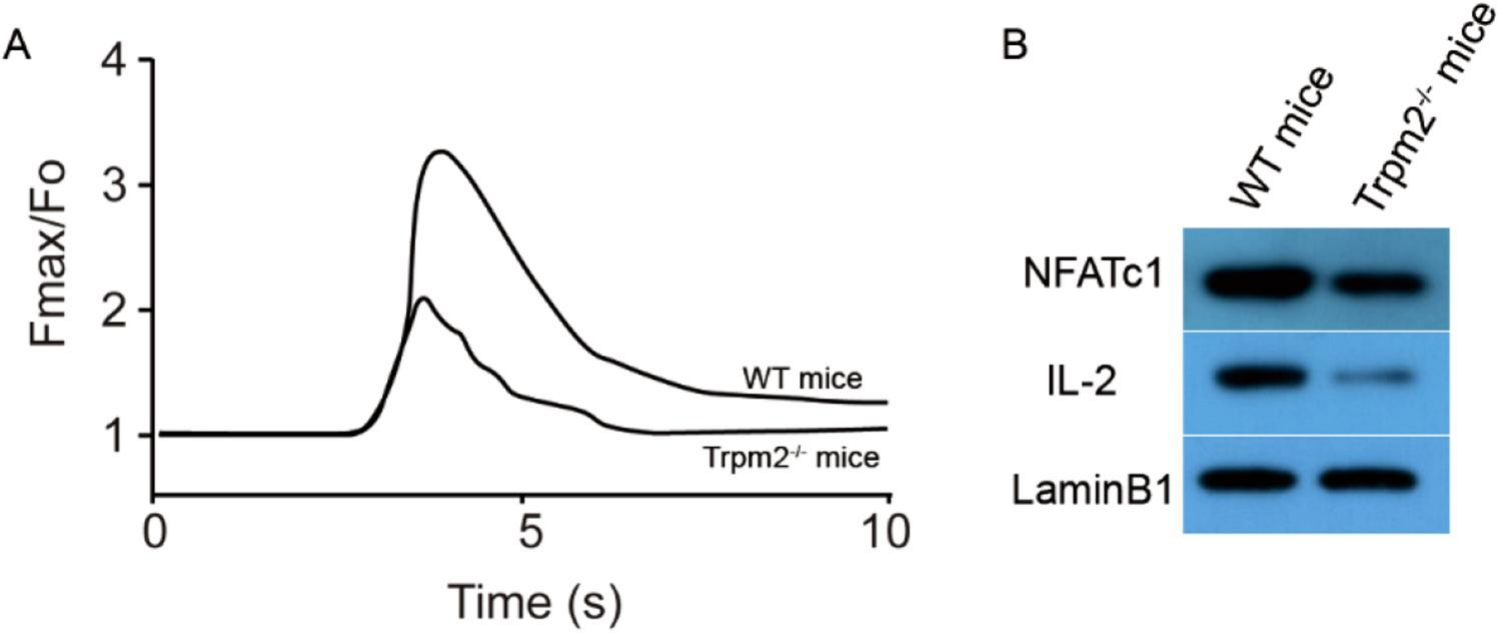
TRPM2 deficiency is associated with attenuated Ca^2+^‐NFAT signaling during T cell activation. (A) Intracellular Ca^2+^ flux in splenic CD4^+^ T cells from WT and TRPM2^−/−^ mice, measured by flow cytometry using Fluo‐4 AM after stimulation with anti‐CD3/CD28 antibodies (5 μg/mL each). The graph shows the mean fluorescence intensity (MFI) over time from one representative experiment of three independent replicates. (B) Nuclear translocation of NFATc1 and IL‐2 production were assessed by Western blot from WT and TRPM2^−/−^ mice.

We next investigated the molecular mechanisms by which TRPM2 may participate in T cell activation. Flow cytometric analysis revealed that TRPM2^−/−^ CD4 + T cells exhibited reduced Ca^2+^ influx upon stimulation compared to WT controls (*p* < 0.001, Figure 3A), accompanied by a significant decrease in NFATc1 nuclear translocation (*p* < 0.01, Figure 3B). This attenuated Ca^2+^‐NFAT signaling was accompanied by decreased IL‐2 production, with TRPM2^−/−^ T cells showing lower IL‐2 levels (*p* < 0.001, Figure 3B). These findings support an association between TRPM2 expression and Ca^2+^‐dependent NFAT signaling during T cell activation.

### 3.6 TRPM2 Deficiency Enhances Treg Responses and Reduces IL‐17 Expression Without Significantly Changing Th17 Frequency

**FIGURE 4:**
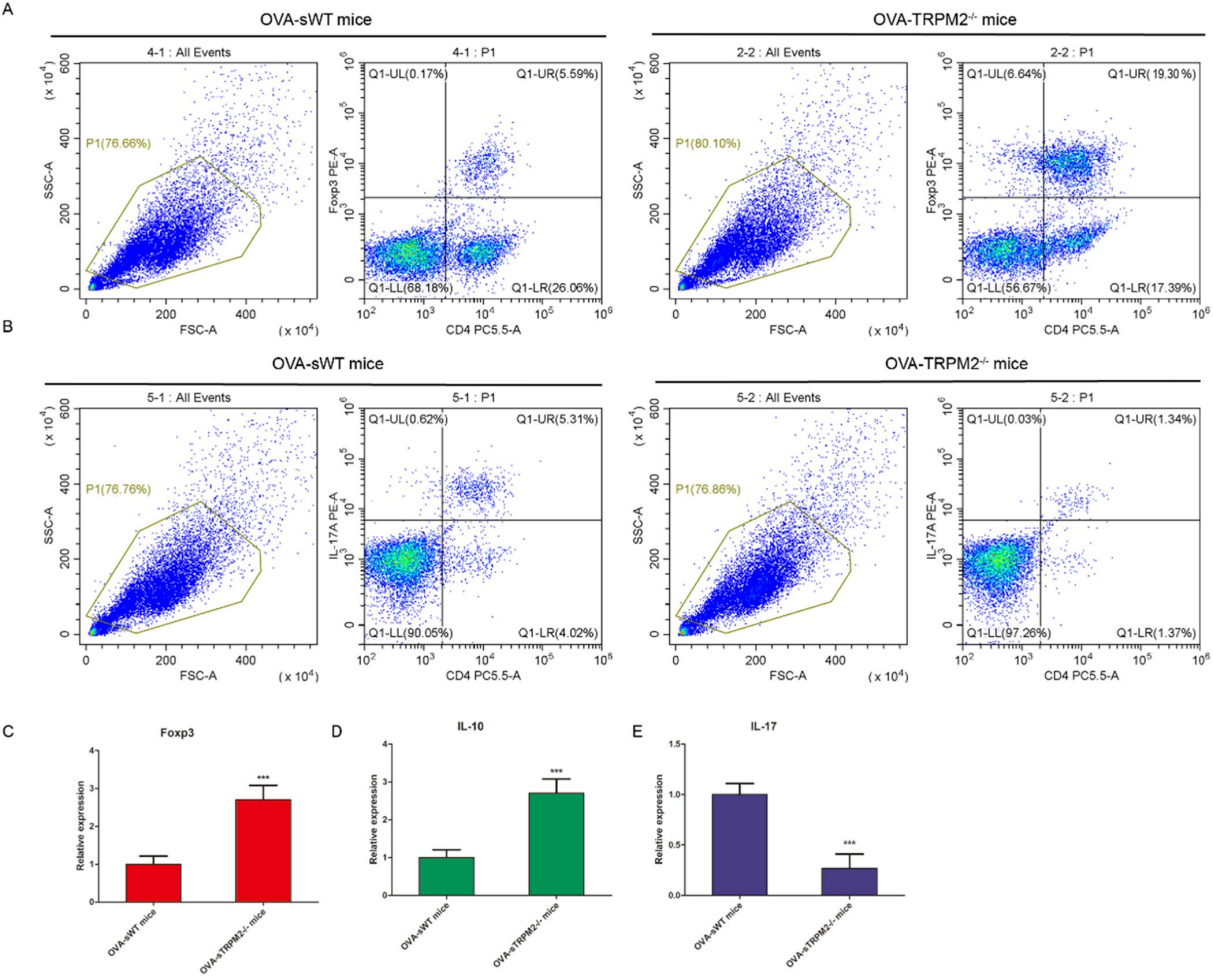
TRPM2 deficiency enhances Treg‐associated anti‐inflammatory markers and reduces IL‐17 expression in allergic rhinitis. (A) The frequency of CD4+Foxp3+ regulatory T cells (Tregs) and (B) CD4 + IL‐17 + T helper 17 cells (Th17) in the indicated groups at 48 h after the final allergen challenge. Th17 frequency showed a downward trend but was not statistically different. (C) qPCR analysis of Foxp3 mRNA (D) IL‐10 and (E) IL‐17 expression in the spleen, normalized to naïve levels. Data are presented as mean ± SEM. ***p < 0.001.

Finally, we examined how TRPM2 affects the balance between regulatory and pro‐inflammatory T cell subsets. Forty‐eight hours after the final challenge, flow cytometric analysis showed that TRPM2^−/−^ mice exhibited increased CD4+Foxp3+ Treg frequency compared to WT controls (*p* < 0.001, Figure 4A). The frequency of CD4 + IL‐17 + Th17 cells showed a downward trend in TRPM2^−/−^ mice, but this difference was not statistically significant (*p* > 0.05, Figure 4B). Molecular analysis revealed corresponding changes in key transcriptional regulators and cytokines: Foxp3 mRNA expression increased 2.7‐fold (*p* < 0.001, Figure 4C). This pattern was accompanied by increased IL‐10 production (2.3‐fold, *p* < 0.01, Figure 4D) and significantly decreased IL‐17 expression in TRPM2^−/−^ mice (*p* < 0.01, Figure 4E). These coordinated changes indicate that TRPM2 deficiency promotes an anti‐inflammatory immune profile primarily through enhanced Treg‐associated responses and reduced IL‐17 expression, whereas suppression of Th17 cell frequency was not statistically confirmed.

## 4 Discussion

The present study investigated the role of the transient receptor potential melastatin 2 (TRPM2) channel in the immunopathogenesis of AR. Employing a genetically modified mouse model deficient in TRPM2, coupled with an established ovalbumin‐induced AR paradigm, the investigation systematically evaluated the impact of TRPM2 ablation on clinical manifestations, inflammatory profiles, and immune cell dynamics. The findings show that TRPM2 deficiency attenuated several hallmark features of AR‐like inflammation, including nasal mucosal inflammation and serum IgE elevation, while shifting immune homeostasis toward enhanced Treg responses and altered calcium‐dependent signaling cascades. These results support the relevance of TRPM2 in AR‐like immunopathology, but they should be interpreted as mechanistically suggestive rather than definitive evidence of direct causality.

The reduction in Ca^2+^ influx after T cell receptor engagement, together with decreased NFATc1 nuclear translocation and IL‐2 synthesis, suggests that TRPM2‐mediated calcium signaling is associated with T cell activation in this model. Mechanistically, TRPM2 functions as a redox‐ and ADPR‐sensitive cation channel, and its activation facilitates the entry of extracellular Ca^2+^, which is required for the subsequent activation of calcineurin and dephosphorylation‐driven nuclear import of NFAT family transcription factors [12, 14]. This TRPM2‐dependent Ca^2+^ influx may integrate upstream signals from reactive oxygen species, linking oxidative stress to immune activation [13]. In the absence of TRPM2, the magnitude and duration of Ca^2+^ signals were reduced, which was accompanied by decreased NFATc1 activation and lower IL‐2 production [15, 16]. Previous reports have suggested that TRPM2 is dispensable for T cell activation under certain in vitro conditions [17], and the present data do not exclude context‐dependent or cell‐type‐specific effects. The observed immune changes may reflect both CD4+ T‐cell‐intrinsic signaling and broader systemic consequences of TRPM2 deficiency, including altered oxidative stress and innate immune activation, which can influence cytokine milieus that favor Treg responses or affect IL‐17 expression [13, 18, 19]. Because rescue experiments, pharmacologic inhibition, and cell‐specific deletion were not included, the Ca^2+^‐NFAT axis should be interpreted as a mechanistically implicated pathway rather than a fully established causal mechanism.

Consistent with these cellular and molecular observations, the attenuation of allergic behavioral responses and tissue pathology in TRPM2‐deficient mice supports the functional relevance of TRPM2‐associated immune regulation in vivo. The observed reductions in eosinophil and monocyte infiltration, as well as preservation of epithelial integrity, are consistent with a model in which TRPM2‐mediated Ca^2+^ entry contributes to the production of pro‐inflammatory cytokines and chemokines that drive leukocyte chemotaxis and tissue remodeling [18]. The behavioral improvements observed in this allergic rhinitis model should also be considered alongside reports from other inflammatory disease contexts, such as colitis and asthma, where TRPM2 has been associated with immune cell activation and tissue‐destructive processes [19, 20]. At the level of humoral immunity, comparative analyses in other allergic and autoimmune models have demonstrated that TRPM2 activity correlates with increased immunoglobulin production and heightened humoral responses, further supporting its possible role as a facilitator of antibody‐mediated immunity [13, 17, 18, 21]. These findings suggest that while TRPM2‐related Ca^2+^ signaling may represent a shared upstream mechanism, downstream effector pathways and clinical manifestations are shaped by the tissue and disease context.

Placing our findings within the contemporary immunological framework further clarifies their significance. The classification of hypersensitivity reactions has evolved considerably since the seminal work of Gell and Coombs. The recent position paper by the European Academy of Allergy and Clinical Immunology (EAACI) proposes a nomenclature adapted to modern needs, distinguishing between IgE‐mediated, non‐IgE‐mediated, and mixed hypersensitivity endotypes [22]. The OVA‐induced AR model employed herein predominantly recapitulates an IgE‐mediated type I hypersensitivity endotype characterized by Th2 skewing, allergen‐specific IgE production, and eosinophilic inflammation. Our data suggest that TRPM2 participates in this endotype by affecting Ca^2+^‐NFAT signaling, Treg‐associated immune regulation, and the magnitude of the IgE response.

The findings may also help interpret aspects of local allergic rhinitis, a phenotype in which allergic inflammation is largely confined to the nasal mucosa and may involve local allergen‐specific IgE production despite limited systemic evidence of atopy. A recent scoping review by Berghi et al. emphasized that local allergic rhinitis remains a challenge requiring close cooperation between allergology and otorhinolaryngology, particularly because diagnosis and management depend on integrating local nasal immune responses with clinical evaluation [23]. In this context, the present findings linking nasal mucosal cytokine expression, OVA‐specific IgE, and Treg‐associated regulation provide a possible experimental framework for understanding local immune dysregulation. However, the current OVA‐sensitized murine model is not equivalent to human local allergic rhinitis, and the relevance of TRPM2 to this phenotype requires dedicated clinical and translational studies.

In summary, TRPM2 deficiency was associated with attenuation of OVA‐induced AR‐like symptoms, reduced nasal inflammation, lower OVA‐specific IgE production, increased Treg‐associated responses, reduced IL‐17 expression, and dampened Ca^2+^‐NFAT signaling. These findings identify TRPM2 as a candidate immunoregulatory pathway in a type 2‐high murine model of AR, rather than a validated therapeutic target. Future studies should extend these observations using chronic, multifactorial, and clinically relevant AR models and should incorporate rescue experiments, pharmacologic inhibition, and cell‐specific TRPM2 ablation to define the spatial and temporal dynamics of its immunomodulatory effects. Such work will be necessary before TRPM2‐targeted strategies can be considered for allergic airway diseases.

## Author Contributions

**Zhenke Huang:** conceptualization, writing – original draft, visualization, validation, methodology, investigation, formal analysis, project administration, data curation, supervision. **Jianping Fan:** conceptualization, methodology, software, formal analysis, data curation, supervision, resources. **Xinxin Shan:** investigation, validation, formal analysis, supervision. **Juntao Su:** data curation, supervision, resources, visualization. **Lixin Zhu:** writing – review and editing, methodology, conceptualization, supervision, resources. **Lijing Peng:** writing – review and editing, conceptualization, methodology, formal analysis, project administration.

## Ethics Statement

The Laboratory Animal Ethics Committee of Shanghai University of Medicine and Health Sciences Affiliated Zhoupu Hospital approved the work.

## Consent

The authors have nothing to report.

## Conflicts of Interest

The authors declare no conflicts of interest.

## Data Availability

The data that support the findings of this study are available from the corresponding author upon reasonable request.
